# Intestinal obstruction due to dual gastrointestinal atresia in infants: diagnosis and management of 3 cases

**DOI:** 10.1186/1471-230X-14-108

**Published:** 2014-06-13

**Authors:** Hua-dong Chen, Hong Jiang, Anna Kan, Li-e Huang, Zhi-hai Zhong, Zhi-chong Zhang, Jun-cheng Liu

**Affiliations:** 1 Pediatric Surgery Department of the First Affiliated Hospital, Sun Yat-sen University, Guangzhou, China; 2 Hepatobiliary Surgery Department of the First Affiliated Hospital, Sun Yat-sen University, Guangzhou, China

**Keywords:** Type I Atresia, Gastrointestinal diaphragm, Congenital, Intestinal obstruction, Infant

## Abstract

**Background:**

Several types of congenital lesions can cause complete or incomplete obstruction of the intestine. Our purpose is to present 3 neonates with dual intestinal type I atresia, i.e., simultaneous obstructive lesions at 2 locations in which the atresia manifested as diaphragm-like tissue.

**Case presentation:**

All 3 cases were female infants ranging in age from 2 to 14 months. The common symptom in all cases was intermittent persistent vomiting. In some cases the vomitus was bilious, and other symptoms included abdominal distention and delayed meconium passage. Prior surgeries at another hospital were unsuccessful at relieving the symptoms in one case. One case had dual lesions in the colon, one dual lesions in the duodenum, and one atresia at both the distal portion of the ileum and the descending colon. Surgical exploration and removal of the lesions at our hospital was successful in all cases, and the infants were discharged in good condition.

**Conclusions:**

Type I atresia can manifest as a diaphragm-like tissue obstructing the continuity of gastrointestinal tract, and in rare cases multiple areas may be present. Base on the intermittent nature of the associated symptoms, diagnosis can be difficult and is often delayed. Physicians should be aware of this condition during the work-up of an infant with persistent intermittent vomiting.

## Background

Several types of congenital lesions can cause complete or incomplete obstruction of the intestines and atresia, failure of the lumen to canalize, accounts for approximately 95% of obstructions [1]. The spectrum of abnormalities causing obstruction includes imperforate and perforate diaphragms of variable thickness within the intestines. Imperforate diaphragms cause complete obstruction, whereas those with central perforations cause incomplete obstruction.

Intestinal atresia can occur anywhere along the GI tract, and the anatomic location of the obstruction and the degree of blockage determine the clinical manifestations [2]. Characteristic clinical features of duodenal atresia are repetitive bilious vomiting, with or without subtle upper abdominal distension. The recognition of partial obstructions may be delayed if the obstruction is of a relatively minor degree. The pertinent signs of jejunoileal atresia include bilious emesis, abdominal distension, jaundice, and failure to pass meconium on the first day of life.

Small intestine atresia accounts for more than 90% of all cases of infants with congenital occlusions, while colon occlusion due to variable septa is rare, with only 6 reports since 1966. Though intestinal atresia usually occurs in a single location (>90% of cases), 6-20% of cases have multiple areas of atresia [3], and more often involve the proximal jejunum. Nevertheless, concurrent type I atresia in both the small intestine and colon have never been reported. We herein present 3 cases of dual intestinal type I atresia located in both the small intestine and colon.

## Case presentations

All three patients had signed informed consent, and the project was approved by the Medical Ethical Committee, The First Affiliated Hospital, Sun Yat-sen University. The details of each GI atresia conditions were described below.

### Case 1

**Figure 1:**
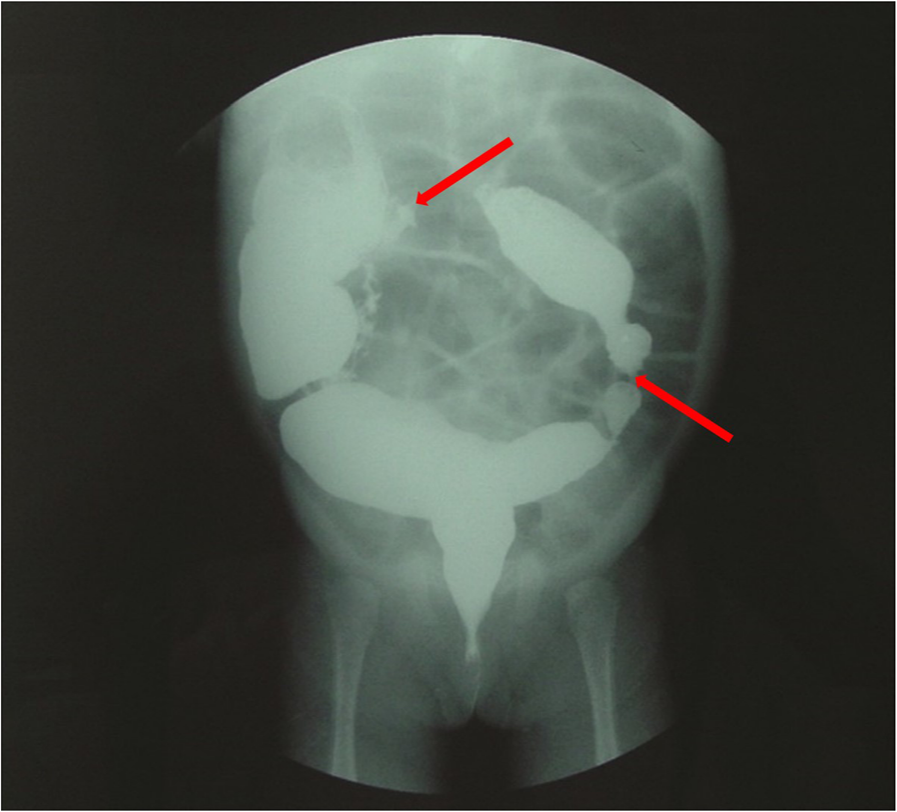
**Case 1.** A 2-month-old female with abdominal distension and difficulty defecating. Barium enema revealed dilation of the colon and 2 areas of atresia (red arrows).

A 2-month-old female presented with abdominal distension and defecation difficulties soon after birth. She was one of premature twins born at 32 4/7 weeks’ gestation. She was admitted to a local hospital for a barium enema, and the results indicated areas of atresia located at both the hepatic and splenic flexures of the colon. Dilation of the colon was noted proximal to the atretic sites (Figure 1). When she was 4 months old, the intestinal obstruction worsened and she was admitted to our institution. We performed an emergent exploratory laparotomy and dilatations were noted midway between the ascending and transverse colon and in the mid-portion of the descending colon. The dilation of the former segment was 3.5 cm in diameter, and that of the later was 2.5 cm, and atretic areas were found distal to both sites of dilation. Therefore, a 2-stage operative correction was planned. First, a colostomy at the hepatic flexure of the colon to was performed to relieve the obstruction. Postoperatively, her defecation improved. Two months later we performed a colectomy at the transverse colon, end-to-end anastomosis of the colon, and colostomy closure. Intraoperatively, areas of atresia which had the appearance of diaphragms were visible at the hepatic and splenic flexures of the colon.

### Case 2

**Figure 2:**
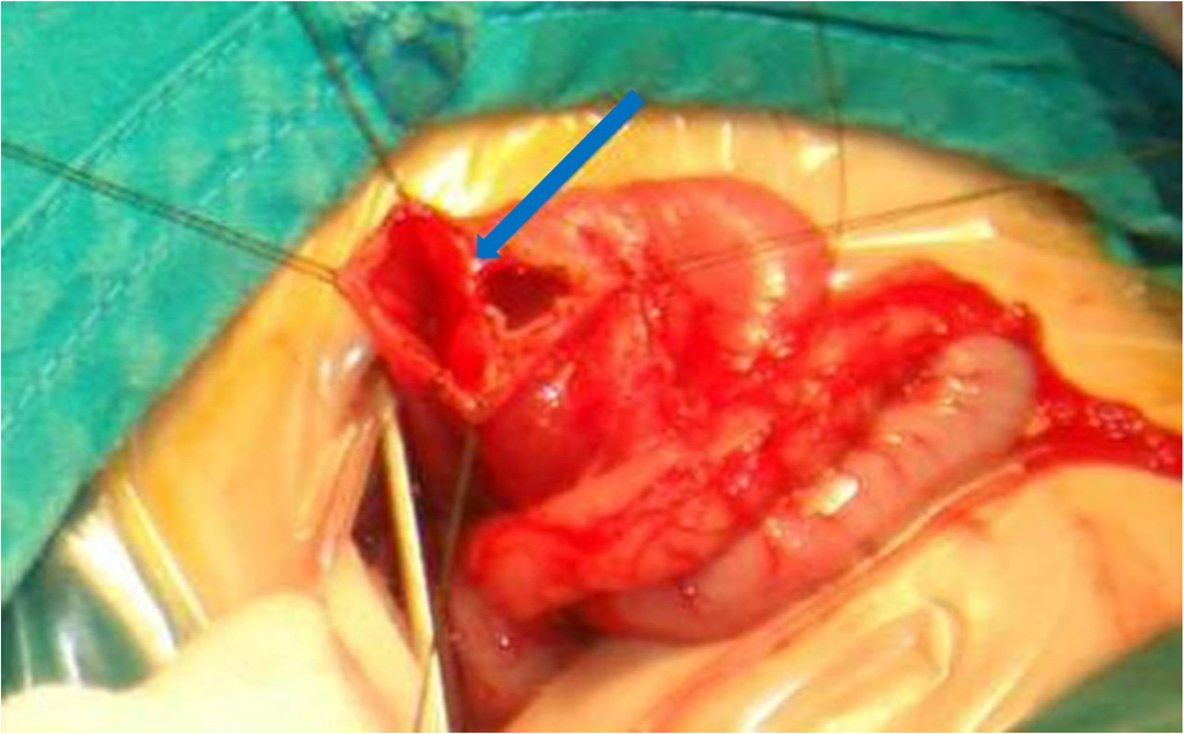
**Case 2.** A 14-month-old female with intermittent vomiting since 3 months after birth. Exploratory laparotomy revealed a duodenal diverticulum at the horizontal segment resulting in a stricture (arrow), which was removed by wedge resection.

A 14-month-old female presented with repeated emesis since 3 months of age. The vomitus was mainly milk with occasional bilious fluid. The vomiting often occurred at night, 2 to 3 times a week. When she was 8 months old the vomiting increased to 2 to 3 times a day, with larger portion of the vomitus containing bile. No evidence of blood in the vomitus or abdominal distension was noted. GI radiography was performed at a local hospital, and obstructions were seen at the third and forth portion of the duodenum. Three surgeries were performed at the local hospital including a Ladd operation and duodeno-duodenostomy; however, her symptoms were not alleviated after these procedures. Hence, she underwent a further exploratory operation at the local hospital, and an obstruction which has the appearance of a diaphragm at the third portion of the duodenum was noted, along with severe adhesions. A proximal duodenostomy, resection of the septum, and enterolysis was performed, and a jejunal nutrition tube was placed. Unfortunately, her symptoms worsened after surgery. She was then admitted to our institution, and reexamination of the GI radiographic studies indicated that there was still an obstruction at the duodenum.Surgical exploration at our hospital revealed significant dilation of the second portion of the duodenum, while the distal intestines were saggy and flat. The third portion of the duodenum was adhered in a U-shape. After releasing the adhesions, a diverticulum was visible at the lateral margin of the mesentery. We removed the duodenum drainage catheter to inject air and extrude the air towards the distal end, and air was noted to pass through. A further attempt was made to place a rubber tube in the third portion of the duodenum, but the placement was inhibited at the junction of the second and third portions of the duodenum. The diverticulum was removed by wedge resection (Figure 2), and the obstructed duodenum was explored. After opening the intestinal lumen, a diaphragm-like area of tissue with a perforation in the center was found at the second and third portion of the duodenum. The diameter of the perforation was 0.3 cm. The segment of the duodenum was incised longitudinally, and the diaphragm-like tissue was completely resected. The duodenal incision was then sutured transversely, and passage of a rubber tube was successful.

Her postoperative course was uneventful, and the symptoms did not recur. At 1 year postoperatively, her growth and development were within the normal range.

### Case 3

**Figure 3:**
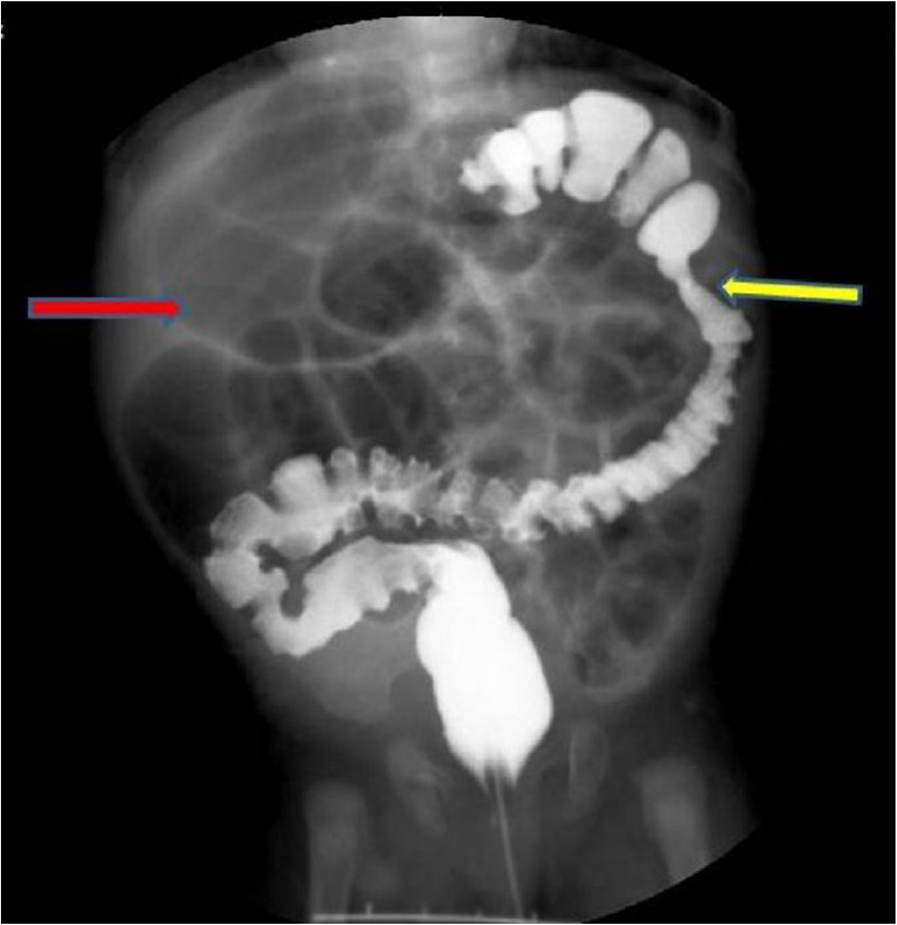
**Case 3.** A 2-month-old female exhibited delayed meconium passage and abdominal distension and vomiting at 10 days after birth. Barium enema revealed an atresia at the splenic side of the colon (yellow arrow), and an air shadow at the ileum (red arrow) indicating expansion.

**Figure 4:**
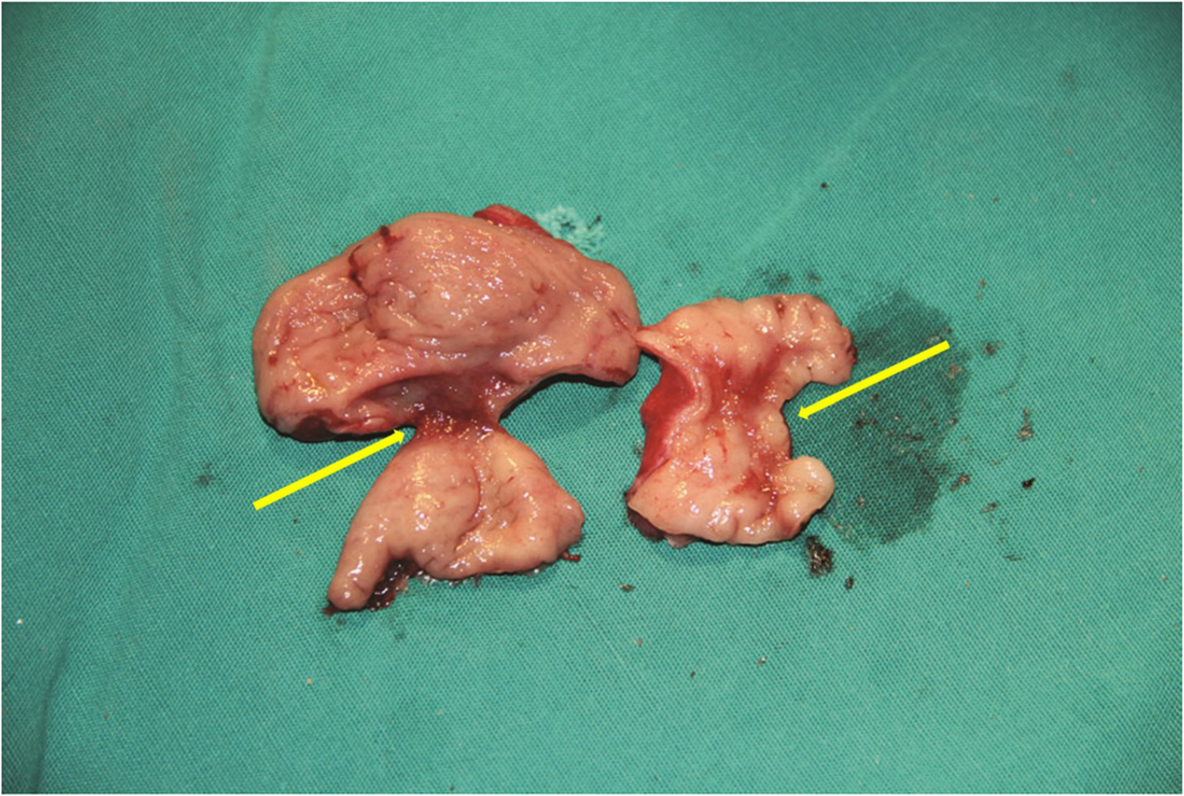
**Case 3.** Examination of the surgical specimens revealed 2 areas of diaphragm-like tissue.

A 2-month-old female presented with abdominal distension and emesis since 10 days after birth. She had no history of delayed meconium passage. She was admitted to our institution, and abdominal radiography showed abdominal distension and a distal digestive tract obstruction was suspected. The initial diagnosis was congenital megacolon, and therefore she was treated with a return-flow enema; however, her symptoms did not improve. After excluding hypothyroidism, a barium enema was performed which revealed an obstruction at the splenic flexure of the colon (Figure 3). The intersection of the transverse and ascending colon was of normal diameter; however, significant dilation was seen at the terminal ileum. With the experience of case 1, we considered the possibility of a dual intestinal obstruction and drafted a detailed protocol before surgery.During surgery, the appendix was found to be adhered to the dilated ileum, and an obstruction about 0.8 cm in length and 0.4 cm in diameter was seen in the terminal ileum. At the intersection of the dilated and obstructed segment of the colon, the intestinal wall was thickened and an obstruction with a diaphragm-like tissue was suspected. The obstructions were resected, and an enterocolostomy and colonic anastomosis was performed. Examination of the 2 surgical specimens revealed diaphragm-like tissue associated with type I atresia (Figure 4).

## Discussion and conclusions

This report presented 3 cases of dual intestinal obstruction in neonates as a result of type I atresia with central perforation of the obstructing diaphragm. As a congenital disease, type I atresia is not a rare abnormality; however, in all of the 3 cases the infants had concurrent atresia along the GI track, which is uncommon among type I atresia patients and complicated arriving at a correct diagnosis.

The differential diagnosis of neonatal upper GI obstruction includes esophageal atresia, malrotation with midgut volvulus, pyloric stenosis, intestinal atresia and stenosis, annular pancreas, preduodenal portal vein, duodenal duplication, foreign body obstruction, Hirschsprung disease, and gastroesophageal reflux [4]. Atresia in the intestines of newborns is a congenital abnormality, which occurs most frequently in the duodenum [5,6] and less frequently in the jejunoileal region [7]. The etiologies of duodenal obstruction and jejunum obstruction are different [1]. On the other hand, congenital colonic atresia is rare [8,9], and should be differentiated from Hirschsprung disease, small left colon syndrome, obturation obstruction, and meconium ileus and meconium plug [9]. Most newborns with intestinal obstruction present with bilious emesis, and in the neonate this should be considered secondary to a mechanical obstruction until proven otherwise and emergent evaluation is warranted [2]. Intestinal atresia is not commonly observed in children, especially those occured at multiple sites [1].

Intestinal atresia may take the form of a transverse diaphragm of tissue with or without a perforation obstructing the continuity of GI tract (type I atresia). The perforation within the diaphragm is usually singular and centrally located within the lumen of the intestine. An intestinal diaphragm may be accompanied with other malformations of the digestive tract such as an annular pancreas [9]. A windsock abnormality is a thin diaphragm that has ballooned distally as a result of peristalsis [9]. Multiple areas of diaphragmatic atresia are only noted in 6-20% of cases [3], and diaphragms located in both the small intestine and colon have never been reported before.

The characteristic clinical feature of almost all intestinal diaphragms is repetitive vomiting. In cases of a perforate diaphragm, the diameter of the opening directly determines the degree of obstruction, and therefore is inversely related to the level of symptoms. Nevertheless, in cases of colonic diaphragmatic atresia, vomiting typically occurs after a full stomach and stools tend to be unformed or thin strip-shaped [10]. In case 2, the patient did not manifest symptoms until 3 months after birth. In that case, successful extrusion of air through the occluded intestine suggests a larger perforation in the diaphragm, which explains the delayed symptoms. These findings may also have contributed to the misdiagnosis prior to admission to our institution. In general, manifestations of colonic type I atresia as seen in cases 2 and 3 are similar to those of congenital megacolon [11,12]. However, when given the routine treatment for congenital megacolon (return-flow enema) the symptoms were not alleviated. Additionally, a large amount of gas had also accumulated in the small intestine, which is not seen in cases of congenital megacolon. A review of 118 cases colonic atresia found the lesion occurred in the ascending colon in 28% of cases, the hepatic flexure in 3%, the transverse colon in 23%, the splenic flexure in 25%, and in the descending and sigmoid in 20% [8].

It is difficult to distinguish type I atresia from other causes of vomiting by clinical manifestations alone. GI radiography and endoscopy are the best methods to diagnose the obstruction before surgery [13]. However, misdiagnosis may still occur. Even intraoperatively, it is challenging to determine the existence of an obstructing diaphragm of tissue by visual observation or palpation [10]. In case 2, a duodenal septum was confirmed intraoperatively and a jejuno-duodenostomy was performed before the patient was admitted to our institution. Unfortunately, obstructive symptoms remained which suggested the presence of a persistent obstruction proximal to the anastomosis. This obstruction was determined to be an intestinal transverse diaphragm during surgery at our hospital. On the other hand, cases 1 and 3 both received barium enema examinations which identified obstructions in the colon. Thus, consideration was given to this specific observation before surgery.

To avoid misdiagnosis, a few key points should be noted before surgery. Surgeons should observe the barium enema being performed which will help them to distinguish abnormalities from normal intestinal movement. In addition, surgeons should fully understand the patient’s prior surgeries and pathogenesis to avoid misdiagnosis of an intestinal diaphragmatic atresia.

## Consent

Written informed consents were obtained from the patients’ parents or guardians for publication of the case report and any accompanying images. Copies of the written consents are available for review by the journal editor.

## Abbreviations

GI: Gastrointestinal.

## Competing interests

The authors declare that they have no competing interests.

## Authors’ contributions

We declare that all the listed authors have participated actively in the study, and all meet the requirements of the authorship. J-CL reviewed the medical records and performed research. H-DC wrote the first draft of the manuscript and performed research. HJ and L-EH performed research. AK, Z-CZ, and Z-HZ managed the literature searches and analyses. All authors read and approved the final manuscript.

## Pre-publication history

The pre-publication history for this paper can be accessed here:

http://www.biomedcentral.com/1471-230X/14/108/prepub
